# Global Alternative Splicing Defects in Human Breast Cancer Cells

**DOI:** 10.3390/cancers13123071

**Published:** 2021-06-20

**Authors:** Jagyeong Oh, Davide Pradella, Yoonseong Kim, Changwei Shao, Hairi Li, Namjeong Choi, Jiyeon Ha, Anna Di Matteo, Xiang-Dong Fu, Xuexiu Zheng, Claudia Ghigna, Haihong Shen

**Affiliations:** 1 School of Life Sciences, Gwangju Institute of Science and Technology, Gwangju 500-712, Korea; jgoh@gist.ac.kr (J.O.); yunseong@gist.ac.kr (Y.K.); njchoi@gist.ac.kr (N.C.); hajiyn@gist.ac.kr (J.H.); xuexiuzheng@gist.ac.kr (X.Z.); 2 Institute of Molecular Genetics “Luigi Luca Cavalli-Sforza”, National Research Council, Via Abbiategrasso 207, 27100 Pavia, Italy; davide.pradella@igm.cnr.it (D.P.); Anna.DiMatteo@igm.cnr.it (A.D.M.); 3 Department of Cellular and Molecular Medicine, University of California, San Diego, La Jolla, CA 92093, USA; c8shao@health.ucsd.edu (C.S.); hairili@health.ucsd.edu (H.L.); xdfu@health.ucsd.edu (X.-D.F.)

**Keywords:** alternative splicing, breast cancer, exon skipping, intrinsically disordered regions

## Abstract

**Simple Summary:**

Aberrant alternative splicing (AS) regulation plays a pivotal role in breast cancer development, progression, and resistance to therapeutical interventions. Indeed, cancer cells can adapt their own transcriptome by changing different AS programs, thus generating cancer-specific AS isoforms involved in every hallmark of cancer. Here, we investigated global AS errors occurring in human breast cancer cells by using RNA-mediated oligonucleotide annealing, selection, and ligation coupled with next-generation sequencing. Our results identified several dysregulated AS events potentially relevant for breast cancer-related biological processes and that provide a better comprehension of the molecular mechanisms that orchestrate the malignant transformation.

**Abstract:**

Breast cancer is the most frequently occurred cancer type and the second cause of death in women worldwide. Alternative splicing (AS) is the process that generates more than one mRNA isoform from a single gene, and it plays a major role in expanding the human protein diversity. Aberrant AS contributes to breast cancer metastasis and resistance to chemotherapeutic interventions. Therefore, identifying cancer-specific isoforms is the prerequisite for therapeutic interventions intended to correct aberrantly expressed AS events. Here, we performed RNA-mediated oligonucleotide annealing, selection, and ligation coupled with next-generation sequencing (RASL-seq) in breast cancer cells, to identify global breast cancer-specific AS defects. By RT-PCR validation, we demonstrate the high accuracy of RASL-seq results. In addition, we analyzed identified AS events using the Cancer Genome Atlas (TCGA) database in a large number of non-pathological and breast tumor specimens and validated them in normal and breast cancer samples. Interestingly, aberrantly regulated AS cassette exons in cancer tissues do not encode for known functional domains but instead encode for amino acids constituting regions of intrinsically disordered protein portions characterized by high flexibility and prone to be subjected to post-translational modifications. Collectively, our results reveal novel AS errors occurring in human breast cancer, potentially affecting breast cancer-related biological processes.

## 1. Introduction

Splicing is an essential process in gene expression in which introns are removed from primary transcripts (pre-mRNAs), thus generating mature RNAs (mRNAs). Essential consensus sequences in the pre-mRNA for the splicing reaction include: 5′ splice-site, 3′ splice-site, branch-point sequence, and the polypyrimidine tract [1,2,3,4]. In the first reaction, the 2′ hydroxyl group of branch point adenosine attacks the 5′ splice-site to form 2′–5′ phosphodiester bond. In the second reaction, the 3′ hydroxyl group of 3′ splice-site attacks the 5′ splice-site to produce a 3′–5′ phosphodiester bond to ligate exons and excise lariat introns [5,6]. The splicing reaction is carried out in the nucleus by the spliceosome, a dynamic and large ribonucleoprotein complex composed of proteins and RNAs [5,6]. Spliceosome contains small nuclear ribonucleoproteins (snRNPs) such as U1, U2, U4, U5, U6 snRNP, their associated proteins, and other non-snRNP protein factors [7].

Alternative splicing (AS) is a post-transcriptional mechanism of gene expression regulation affecting nearly all human protein-coding genes [8,9]. From a single pre-mRNA, AS generates multiple mature mRNAs to produce protein isoforms with different structure, function, stability or cellular localization [10]. Diverse AS modalities include exon skipping (also known as cassette exon), alternative 5′ and 3′ splice site selection, intron retention, and mutually exclusive exons. Precise expression and coordination of specific AS events play key roles to establish fundamental properties during developmental processes, such as angiogenesis [11], neurogenesis [12], immune system homeostasis and differentiation [13], erythropoiesis [14], and in response to extra-cellular signals [15]. The relevance of AS is also underscored by the fact that aberrantly AS switches have been observed in several human diseases, such as cardiovascular diseases [16], diabetes [17], Alzheimer’s disease [18], and cancer [19].

Notably, increasing evidence of a causative role of aberrant AS in cancer has been provided [20]. Indeed, the identification of tumor-specific AS variants has supported the idea that the fidelity of the splicing reaction is lost during tumorigenesis and cancer progression [21]. Remarkably, several genes undergo aberrant AS regulation and generate AS isoforms involved in key aspects of tumor cell biology, such as proliferation, apoptosis, evasion from growth suppressors, angiogenesis, invasion, and metastasis [22]. A global dysregulation of AS programs has also been observed in different processes occurring during development and cancer progression, including EMT (epithelial-to-mesenchymal transition) [23], an important mechanism by which cancer cells acquire migratory and invasive capabilities becoming metastatic. Breast cancer is the most frequent cancer type diagnosed and the second cause of death in women [24]. Global transcriptomic studies and high-throughput sequencing technologies have allowed the identification of several AS errors occurring in breast cancer cells [25]. Notably, several AS events contribute to breast cancer metastasis and resistance to chemotherapeutic interventions [26]. Identification of AS defects occurring in breast cancer cells thus represents an attractive source for novel targets for therapeutic interventions.

RNA-mediated oligonucleotide annealing, selection, and ligation coupled with next-generation sequencing (RASL-seq) uses a pool of primer pairs that are specific to exon junction to detect ~5600 known AS events that are conserved between human and mice [27]. As opposed to completely unbiased profiling of AS by RNA-seq, RASL-seq focuses on annotated targets without allowing de novo discovery of AS events. However, RASL-seq is robust in quantitatively determining expression differences of mRNA isoforms [27,28]. RASL-seq can be used to compare and characterize AS programs in different cells or patient samples [27,28,29]. Importantly, numerous annotated AS events related to cancer progression are included in RASL-seq categories.

To identify global AS defects in human breast cancer cells, here we performed RASL-seq of MCF10A and MCF7 cells, representing non-tumorigenic and cancer human breast cell lines, respectively [30,31]. We demonstrated that RASL-seq represents a valuable tool for the identification of global AS defects occurring in breast cancer cells. In addition, we take advantage of the Cancer Genome Atlas (TCGA) database to analyze the expression profiles of our identified AS events in a large number of non-pathological and tumoral breast specimens. Importantly, this in silico analysis was also validated by RT-PCR in a restricted number of human normal and breast cancer samples. Furthermore, in order to assess the functional relevance of AS errors occurring in breast cancer cells, we analyzed the effect of cassette exon inclusion or skipping at the protein level. Interestingly, our analysis showed that a number of the AS exons with altered expression in breast tumors specimens versus non-pathological adjacent tissues do not encode for known functional domains but instead encode for amino acids constituting regions of intrinsically disordered protein portions characterized by high flexibility and prone to be subjected to post-translational modifications.

## 2. Materials and Methods

### 2.1. Cell Culture, RNA Extraction, and RT-PCR

MCF10A (American Type Culture Collection (ATCC), CRL-10317TM) and MCF7 (American Type Culture Collection (ATCC), HTB-22^TM^) cells were cultured in RPMI medium (HyClone, Logan, UT, USA, Cat: SH30027.01) supplemented with 10% fetal bovine serum (FBS, HyClone, Logan, UT, USA, Cat: SH30084.03), 2 mM glutamine, 100 U/mL penicillin, and 100 μg streptomycin at 37 °C in a 5% CO_2_ incubator. Total RNAs were extracted using the RixoEX reagent (GeneAll, Seoul, Korea, Cat: 301-001) according to the manufacturer’s instruction. Reverse transcription was performed using M-MLV reverse transcriptase (elpis, Daejeon, Korea, Cat: EBT-1028) or Superscript IV reverse transcriptase (Invitrogen, Carlsbad, California, USA, Cat: 18090010). Then, 0.5–1 μg RNAs were used to synthesize cDNA, and then 0.5 μl cDNA was used for PCR amplification using GAPDH as a control. RNA extracts of breast tumor specimens and non-pathological adjacent tissues were purchased from Ambion (#AM7221 and #AM6952). Primers used in the RT-PCR analysis are listed in Table S1.

### 2.2. Alternative Splicing Analysis with RASL-Seq

**Figure 1:**
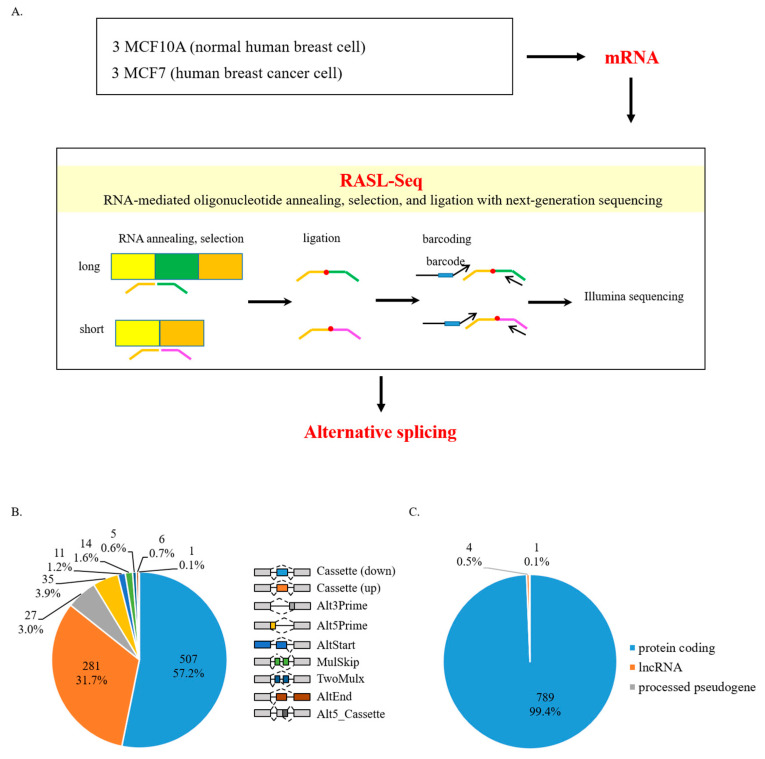
Pre-mRNAs undergo altered AS in human breast cancer cells. (**A**) Schematic representation of the RASL-seq technique using RNA extracted from human breast cancer cells (MCF7) and normal breast cells (MCF10A). (**B**) Pie chart showing different AS events from RASL-seq results. All events listed are drawn in the right. (**C**) Gene distribution of our RASL-seq results.

A pool of oligonucleotides was designed to detect 5530 AS events in RASL reaction as previously described [27] (Figure 1A). Cassette exon included and excluded mRNA isoforms of one gene were detected with two oligonucleotide sets. Total mRNAs were hybridized with the mixture of oligonucleotides and selected with biotin-labeled oligo dT. Two nearby oligos were then ligated and barcoded for high-throughput sequencing with Illumina HiSeq 2500 apparatus. A minimum of 5 read counts in all biological triplicates were filtered for splicing events. The criteria in ratio change of at least 2 and *p*-value < 0.05 were used to filter AS changes. Gene enriched in up-, down- and non-differentially (ndiff) regulated AS events in human breast cancer cells are listed in Table S2.

### 2.3. Gene Ontology (GO) Analysis

GO analysis of enriched functions in up- or down-regulated splicing was performed using EnrichR (https://maayanlab.cloud/Enrichr/) (accessed on 14 May 2021) [32]. AML1/AMP19 fusion term was excluded from the GO analysis.

### 2.4. Splicing Analysis of Transcriptomic Data

Splicing profiles of aberrant AS events, identified by RASL-seq in MCF10A/MCF7, were analyzed in tumor and normal specimens from the TCGA-BRCA level 3 dataset by using the TCGA SpliceSeq web-tool (http://projects.insilico.us.com/TCGASpliceSeq) (accessed on 15 December 2020) [33], a web-based resource known to provide Percent Splice-In (PSI) values for annotated splicing events in specimens of the TCGA. The classification of tumor cohort in basal vs. luminal (luminal A and luminal B considered together) was retrieved by Xena Browser (https://xenabrowser.net/) (accessed on 14 May 2021) according to the latest classification [34].

### 2.5. Prediction of Intrinsically Disordered Regions (IDRs)

Protein domains and the fraction of disordered residues in AS events were retrieved by using the Vertebrate Alternative Splicing and Transcription Database online-tool (VastDB; http://vastdb.crg.eu/wiki/Main_Page) (accessed on 15 December 2020) [35]. For each validated AS event, the disordered rate of the alternative exon (A) and the adjacent constitutive exons (C1 and C2) were collected.

Disordered protein region predictions were also validated by using PONDR web-tool (http://www.pondr.com) (accessed on 15 December 2020) [36,37]. The longest protein variant for each validated AS event was analyzed by using various algorithms optimized for different types of disordered regions (VLXT, SL1_XT, CAN_XT, VL3, and VSL2).

### 2.6. Analysis of Protein-Protein Interaction Networks

Human protein–protein interaction (PPI) networks were obtained from the STRING database (version 11.0; https://string-db.org/) (accessed on 15 December 2020) [38]. The STRING database collects, scores, and integrates PPI information from different available sources [38]. Association networks were generated by considering the following categories: (i) Biochemical/genetic data (“experiments”); (ii) previously curated pathway and protein-complex knowledge (“database”).

### 2.7. Correlation of Drug Sensitivity and Transcript-Specific Expression of AS Isoforms

Drug response data of 41 breast cancer cell lines were retrieved through the Genomics of Drug Sensitivity in Cancer Project (https://www.cancerrxgene.org) (accessed on 14 May 2021) [39]. The percentage (%) of transcripts including the indicated AS event was obtained by the Cancer Cell Line Encyclopedia (CCLE) database [40]. To calculate the % of transcripts in which a specific AS exon is present, isoform-level expression in TPM (transcript per million)-quantified using the RSEM method-of transcripts including the exon of interest were summed and then divided for the total TPM values of isoforms. Only isoforms in which at least one constitutive exon at 5′ and one at the 3′ of the analyzed exon are present have been considered. AUC (area under the dose-response curve) values of paclitaxel (GDSC2, SANGER), docetaxel (GDSC2, SANGER), 5-fluorouracil (GDSC2, SANGER), tamoxifen (GDSC2, SANGER), and gemcitabine (GDSC2, SANGER) of available cancer cell lines were matched with the % of transcripts including the AS exon of the CCLE project for each cell line.

## 3. Results

### 3.1. AS Defects in Human Breast Cancer Cells

In order to identify aberrant AS events occurring in human breast cancer cells, we performed RASL-seq in non-tumorigenic human mammary epithelial cells (MCF10A) and human breast cancer cells (MCF7) (Figure 1A). From 5530 available events detectable by RASL-seq technology in the human genome, we obtained 1243 events with ≥5 read counts and *p*-value < 0.05. Among them, 332 AS events had a ratio increase of at least 2, 555 AS events had a ratio decrease of at least −2. Most of these events with significant ratio changes were cassette exons (Cassette), with 281 increased and 507 decreased events (Table S2) (Figure 1B). In addition, we were also able to observe 27 alternative 3′ splice site (Alt3Prime), 35 alternative 5′ splice site (Alt5Prime), 11 alternative transcription initiation (AltStart), 5 multi exon skipping (TwoMulx), 14 mutually exclusive exon (MulSkip), 6 alternative transcription termination (AltEnd), and 1 alternative 5′ cassette exon (Alt5_Cassette) (Table S2) (Figure 1B). We further identified that most of the genes with altered AS were protein-coding genes (789 genes, 99.4%), whereas only a small portion of genes were pseudogenes (4 genes, 0.5%) or lncRNAs (1 gene, 0.1%) (Figure 1C) (Table S3). These results suggest that human breast cancer cells are characterized by substantially aberrant AS events compared to normal breast cells.

### 3.2. GO Analysis Identifies Functions Related to Oncogenesis in Genes with Altered AS Profiles in Breast Cancer Cells

**Figure 2:**
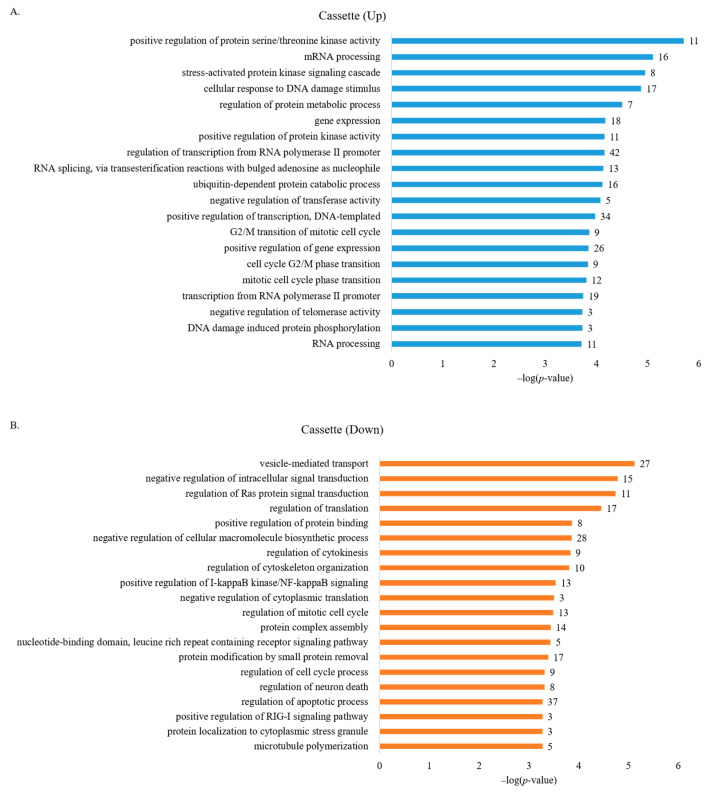
GO analysis of genes with aberrant AS regulation in human breast cancer cells identified several biological processes related to cancer progression. (**A**) GO analysis of genes enriched in up-regulated AS cassette exons. (**B**) GO analysis of genes enriched in down-regulated AS cassette exons.

We next applied GO analysis to understand the roles of genes with aberrantly regulated (up and down) AS cassette exons in human breast cancer cells. Figure 2A shows that functions of genes with increased cassette exon splicing were enriched in positive regulation of protein serine/threonine kinase activity, mRNA processing, stress-activated protein kinase signaling cascade, cellular response to DNA damage stimulus, regulation of protein metabolic process, and gene expression (Table S4). The functions of genes with decreased cassette exon splicing were enriched in vesicle-mediated transport, negative regulation of intracellular signal transduction, regulation of Ras protein signal transduction, regulation of translation, negative regulation of cellular macromolecule biosynthetic process, and regulation of cytokinesis (Figure 2B, Table S4). Among these processes, involvements of mRNA processing, transcription, and cell cycle are enriched, suggesting that AS alterations in breast cancer cells may affect gene expression at different levels that implicate the involvement of transcriptional, post-transcriptional, and epigenetic programs. Importantly, different processes identified in up- or down-regulated AS events are directly linked to oncogenesis and cancer progression.

### 3.3. Validation of Aberrantly Regulated AS Events in Breast Tumor Cell Lines

**Figure 3:**
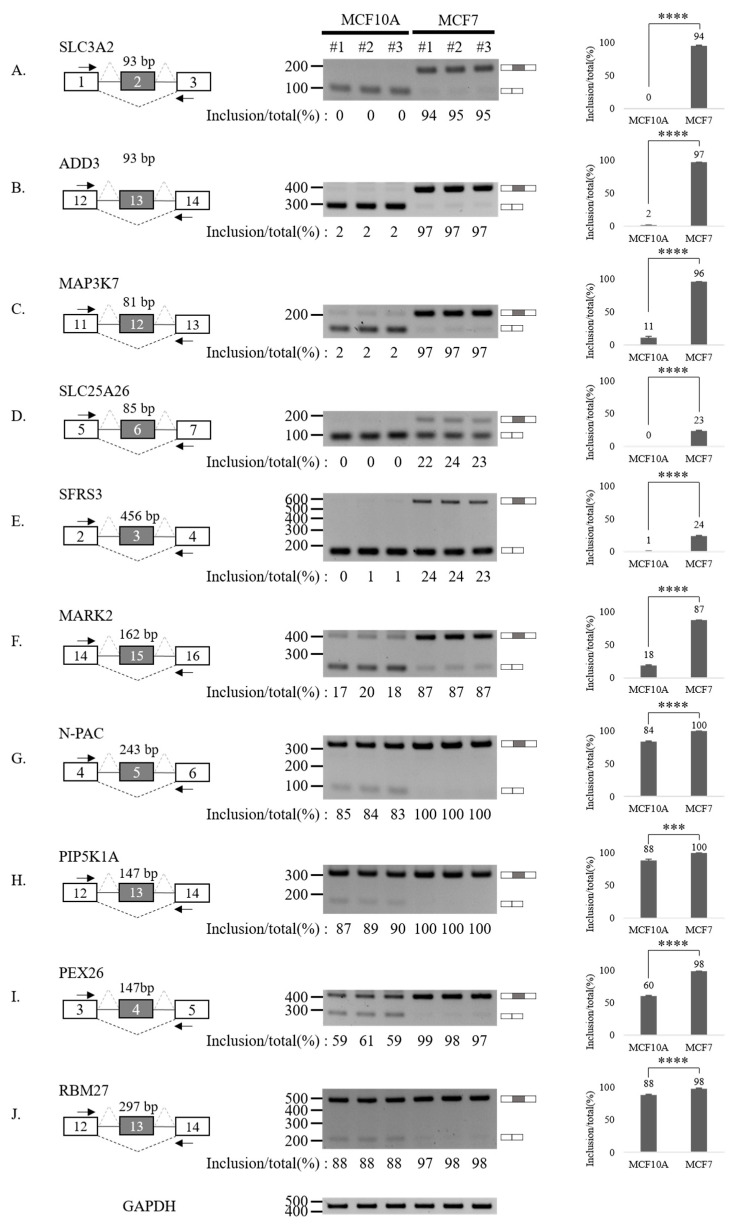
Validation of AS up-regulated cassette exons in human breast cancer cells. (**A**–**J**) (Left) For each gene, the schematic representation of the genomic region containing the AS cassette exon is shown. Exon numbers are shown in the gray boxes. The length of AS cassette exons is shown. Inclusions of cassette exons are shown with dotted grey lines. Skipping events are shown with dotted black lines. Arrows indicate primers used in RT-PCR. (Middle) RT-PCR analysis (in triplicated) of the AS profile of cassette exons was performed by using RNA extracted from human breast cells. Quantitation results are shown at the bottom of each gel. (Right) Statistical analysis graphs of RT-PCR. Error bars = standard deviation (SD) calculated from three independent experiments. **** *p* < 0.0001, *** *p* < 0.001. Uncropped figures are shown in Supplementary Figure S7.

**Figure 4:**
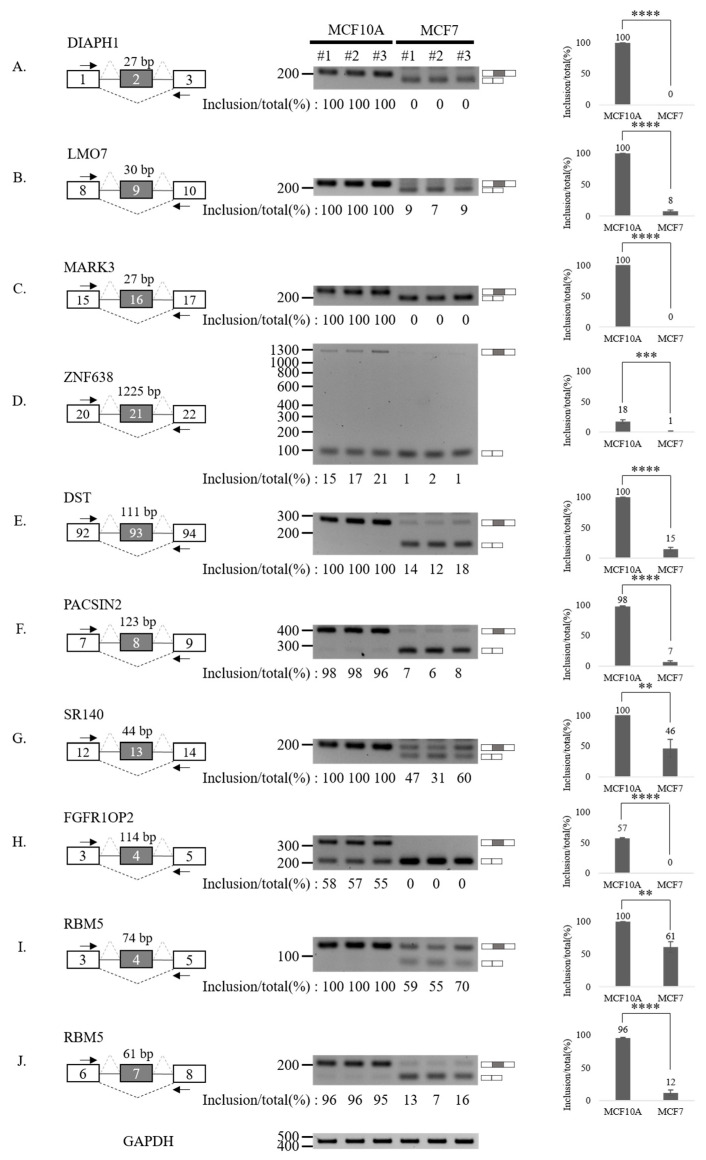
Validation of AS down-regulated cassette exons in human breast cancer cells. (**A**–**J**) (Left) For each gene, the schematic representation of the genomic region containing the AS cassette exon is shown. Exon numbers are shown in the gray boxes. The length of AS cassette exons is shown above the exon. Inclusions of cassette exons are shown with dotted grey lines. Skipping events are shown with dotted black lines. Arrows indicate primers used in RT-PCR. (Middle) RT-PCR analysis (in triplicated) of the AS profile of cassette exons was performed by using RNA extracted from human breast cells. Quantitation results are shown at the bottom of each gel. (Right) Statistical analysis graphs of RT-PCR. Error bars = standard deviation (SD) calculated from three independent experiments. **** *p* < 0.0001, *** *p* < 0.001, ** *p* < 0.01. Uncropped figures are shown in Supplementary Figure S8.

To validate our RASL-seq results, we performed RT-PCR analysis of 20 altered AS cassette exons (Figure 3 and Figure 4). In particular, we selected 10 events among AS cassette exons that are up-regulated in breast cancer cells, and 10 events from AS cassette exon highly down-regulated in tumor cells. We validated increases (Figure 3A–J) and decreases (Figure 4A–J) in exon cassette inclusion. Genes with significant AS changes of cassette exons in tumor vs. normal cell lines (Figure 3 and Figure 4) are shown in Table S5. Interestingly, several genes have an annotated role in breast cancer progression and outcome of disease in cancer patients (Table S5, column 8).

Collectively, our results indicate that RASL-seq has a high validation rate and represents a valuable tool to identify global aberrantly expressed AS events in cancer cells.

Notably, MCF7 cells are known to express estrogen receptors (ER-positive) [41]. To investigate if estrogen receptor expression could affect our identified AS changes, we validate the selected 20 events in additional breast cancer cell lines ER-positive (T47D) and triple-negative breast cancer cells (TNBC) (MDA-MB-231 and BT549) [41].

As shown in Figure S1, we were able to confirm 13/20 aberrantly regulated AS events in another ER-positive breast cancer cell line (T47D). Among these, five AS changes (*RBM27* exon 13; *PIP5K1A* exon 13; N-PAC exon 5; *MARK2* exon 15, and *PEX26* exon 4) were also observed—to different extents—in MDA-MB-231 and BT549 cell lines, thus suggesting their independence from the ER status of the breast tumor, whereas for seven events (*ADD3* exon 13, *MAP3K7* exon 12, *MARK3* exon 16, *PACSIN2* exon 8, *FGFR1OP2* exon 4, and, with a less marked splicing change, *DIAPH1* exon 2, *LMO7* exon 9), an aberrant AS profile was found only in ER-positive breast cancer cells (Figure S1).

Moreover, to assess the importance of genes harboring AS errors, we analyzed protein-protein interaction (PPI) networks of proteins harboring the 20 validated cassette AS exons. Remarkably, identified PPI networks reveal direct interactions of the analyzed proteins with factors involved in malignant transformation, such as RHOA (DIAPH1 interactor), PIK3CA (FGFR1OP2 interactor), and PRKCI (MARK2 interactor) (Figures S2 and S3).

Collectively, our results suggest that human breast cancer cells express highly specific AS isoforms of genes involved in cancer-related biological processes compared to non-tumorigenic breast cells.

### 3.4. Validation of Aberrantly Regulated AS in the TCGA-BRCA Dataset and in Breast Tumor Specimens

**Figure 5:**
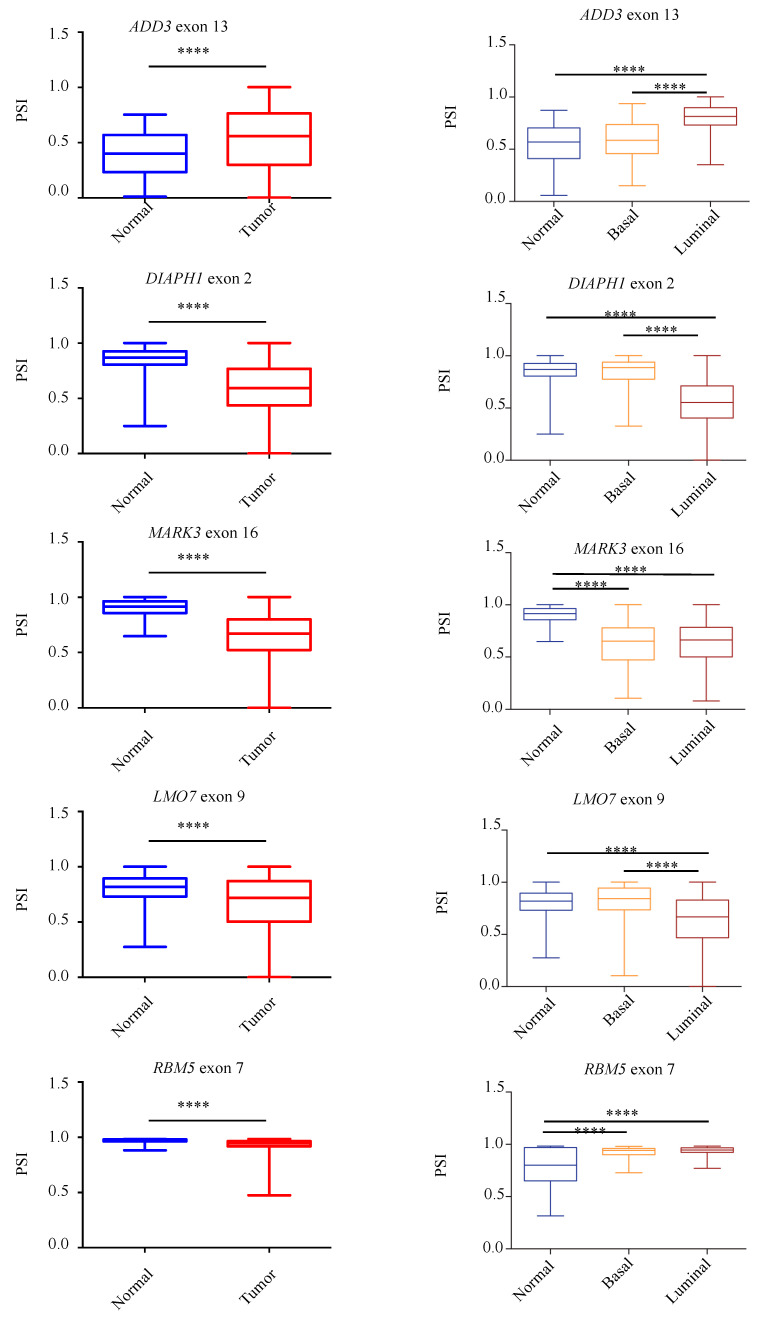

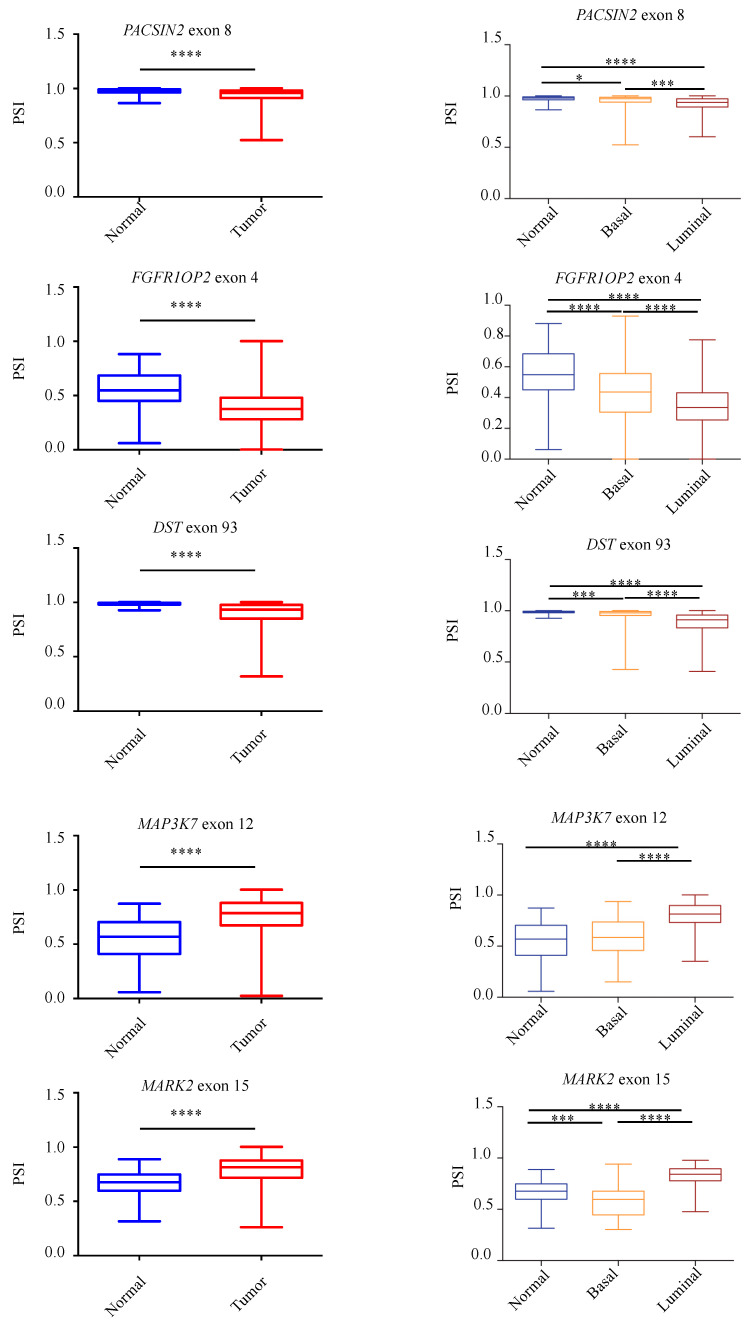
Validation of RASL-seq AS events in breast cancer specimens and in non-pathological tissues. Analysis, by using the TCGA SpliceSeq online tool, of the splicing pattern of *ADD3* exon 13, *PACSIN2* exon 8, *DIAPH1* exon 2, *FGFR1OP2* exon 4, *MARK3* exon 16, *DST* exon 93, *LMO7* exon 9, *MAP3K7* exon 12, *RBM5* exon 7, and *MARK2* exon 15 in normal and primary tumor specimens from the TCGA-BRCA dataset (left) and in normal and primary tumor specimens divided for their histopathological state (basal or luminal) (right). PSI = Percent Splice-In. **** *p* < 0.0001, *** *p* < 0.001, * *p* < 0.05.

To further assess the presence of the identified aberrant AS events in tumors compared to normal breast tissues, we take advantage of the Cancer Genome Atlas (TCGA-BRCA) [34] to compare a large number of non-pathological samples with primary breast tumors. In particular, we analyzed the splicing pattern of 10 RASL-seq identified and validated events by using the TCGA SpliceSeq web-tool (http://projects.insilico.us.com/TCGASpliceSeq; accessed on 14 May 2021) [33]. As shown in Figure 5, we confirmed an aberrant increase of cassette exon skipping in tumor specimens for *PACSIN2* exon 8, *DIAPH1* exon 2, *FGFR1OP2* exon 4, *MARK3* exon 16, *DST* exon 93, *LMO7* exon 10, and *RBM5* exon 7, whereas *ADD3* exon 13, *MAP3K7* exon 12, and *MARK2* exon 15 were preferentially included in tumor specimens. Noteworthily, for the majority of the events, luminal tumors (A and B subtypes) showed a higher difference in the PSI with respect to basal ones when compared to normal tissues (Figure 5). Importantly, we were also able to confirm, by RT-PCR, the aberrant AS regulation of *PACSIN2*, *DIAPH1*, *MARK3*, *ADD3*, *MAP3K7*, and *MARK2* in RNA extracts from two human tumor breast cancer samples tissues and two non-pathological tissues (Figure S4).

Collectively, our results further support the notion that an extensive AS dysregulation takes place in breast cancer. Notably, our analysis identifies novel perturbed AS events in genes (*PACSIN2* exon 8, *DIAPH1* exon 2, *MARK3* exon 16, *ADD3* exon 13, *MAP3K7* exon 12, and *MARK2* exon 15) involved in different cancer-related biological processes such as migration, apoptosis, DNA-damage, cytoskeleton organization, and proliferation.

### 3.5. Aberrantly Expressed AS Exons Encode for Intrinsically Disordered Regions (IDRs)

To determine the functional relevance of aberrantly expressed AS exons in breast cancer cells and tissues, we take advantage of the Vertebrate Alternative Splicing and Transcription Database (VastDB; http://vastdb.crg.eu/wiki/Main_Page; accessed on 14 May 2021) [35].

We found that five genes (*SLC25A26*, *PEX26*, *RBM27*, *FGFR1OP2*, and *RBM5*) contain cassette exons encoding for protein’s functional domains (Table S5, column 7) in the VastDB database, which comprises PFAM and PROSITE annotations [35].

However, we focused our attention on *PACSIN2*, *DIAPH1*, *MARK3*, *ADD3*, *MAP3K7*, and *MARK2* cassette exons as these events were validated in both normal and cancer cell lines and in non-pathological and tumor breast specimens (Figure S4).

**Figure 6:**
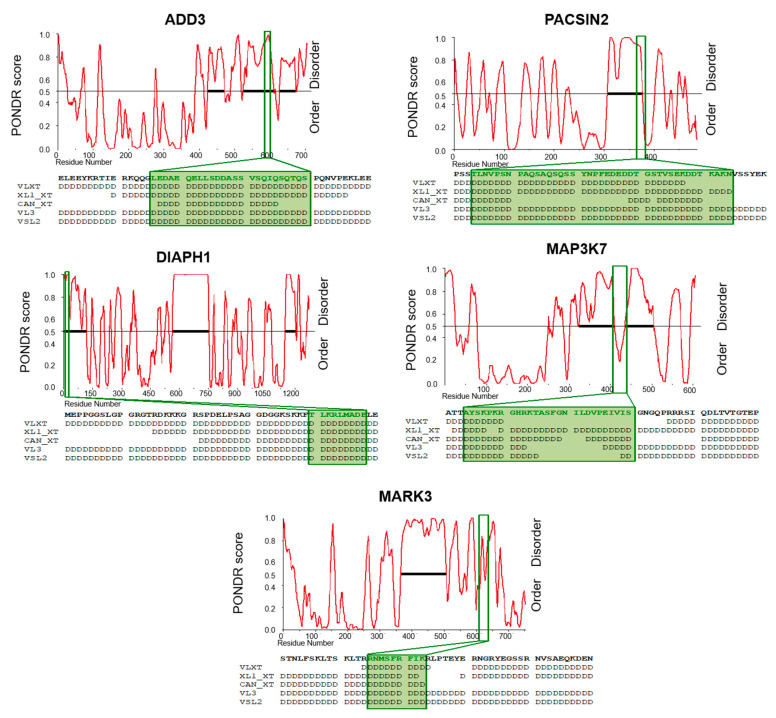
Prediction of intrinsically disordered regions (IDRs) in the validated aberrantly expressed exons in breast tumors. IDRs were predicted by using the PONDR web tool. Graphical representation, using only the VL-XT algorithm, of ADD3, PACSIN2, DIAPH1, MARK3, and MAP3K7 PONDR Scores for each residue. The disorder threshold (0.5) is shown as the black line. Positions of aberrantly expressed AS exons are indicated by green boxes. Below each representation, the amino acid sequence of each AS exon is reported in bold. Adjacent residues are also shown. The disordered residues predicted by different algorithms (VLXT, XL1_XT, CAN_XT, VL3 and VSL2) are indicated with “D”.

Interestingly, dysregulated AS cassette exons do not encode for any predicted functional domain. Nevertheless, we found that these regulated exons encode for amino acids overlapping with intrinsically disordered regions (IDRs) (Table S6). IDRs are highly flexible regions, frequently enriched with charged/polar amino acids and depleted of hydrophobic residues that connect structured protein domains [42]. Importantly, these sequences do not mediate co-operative folding and lack a unique three-dimensional structure, thus providing an extreme flexibility that could favor conformational heterogeneity and post-translational modifications [43]. To further confirm the presence of IDR in the protein regions encoded by breast-cancer-associated AS events, we analyzed ADD3, PACSIN2, DIAPH1, MARK3, and MAP3K7 protein sequences with the PONDR web tool, a predictor of natural disordered regions [36,37]. As shown in Figure 6, the amino acids encoded by these exons are located in IDR predicted by different PONDR algorithms (Figure 6).

### 3.6. Expression of Aberrantly Expressed IDR-Encoding AS Exons Is Associated to Chemotherapeutic Sensitivity in Breast Cancer Cell Lines Databases

**Figure 7:**
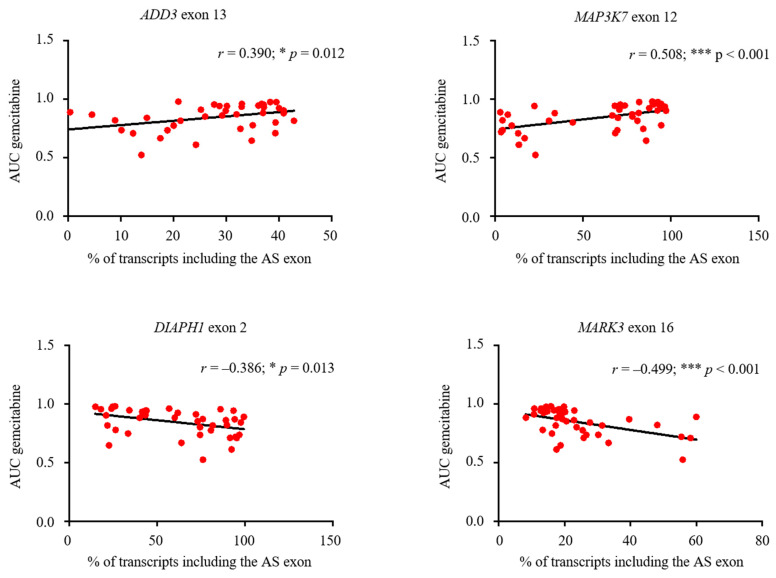
Correlation of IDR-encoding AS events and gemcitabine sensitivity in breast cancer cells. *ADD3* exon 13, *MAP3K7* exon 12, *DIAPH1* exon 2, and *MARK3* exon 16 containing isoforms (% of transcripts) and gemcitabine sensitivity (AUC, area under the dose–response curve) correlation in 41 breast cancer cell lines. Pearson coefficient (r) and linear regression are also shown. *** *p* < 0.001; * *p* < 0.05.

Finally, to explore the potential relevance of IDR-encoding AS exons for chemotherapeutic sensitivity or resistance, we combined drug sensitivity data of classical compounds (paclitaxel, docetaxel, 5-fluorouracil, tamoxifen, and gemcitabine) used for the treatment of breast cancer patients [44,45,46,47] with transcript expression data of 41 breast cancer cell lines retrieved through the Genomics of Drug Sensitivity in Cancer Project and the Cancer Cell Line Encyclopedia (CCLE) database, respectively (Table S7). As shown in Figure 7, we observed a significantly positive correlation between *ADD3* exon 13 (*r* = 0.390; *p* = 0.012) and *MAP3K7* exon 12 (*r* = 0.508; *p* < 0.001) inclusion levels (% of transcripts including the AS exon) and gemcitabine sensitivity (measured as AUC, area under the dose–response curve); whereas a significantly negative correlation is present between the inclusion levels of *DIAPH1* exon 2 (*r* = −0.386; *p* = 0.013) and *MARK3* exon 16 (*r* = −0.499; *p* < 0.001) and gemcitabine sensitivity values (Figure 7; Figure S5). No correlation between any drug treatment and *PACSIN2* exon 8 levels was found (Figure S6).

Collectively, our results indicate that AS profiles of IDR-encoding exons altered in breast cancer cells are potentially linked to chemotherapeutic sensitivity, in particular to the gemcitabine treatment, thus further suggesting the relevance of these events for breast cancer biology.

## 4. Discussion

Alternative splicing (AS) plays key roles in breast tumorigenesis and cancer progression [48,49]. The relevance of AS in cancer cells is highlighted by the fact that AS errors affect thousands of genes involved in different cancer-related biological processes, thus directly impacting most of the “hallmarks of cancer” described by Hanahan and Weinberg [21]. Indeed, AS dysregulation has emerged itself as a “hallmark”, frequently altered in cancer cells [50,51].

By using RASL-seq technology, we corroborated the widespread AS dysregulation occurring in breast cancer cells. In particular, our RASL-seq, which was able to detect 1243 annotated AS events, identified 887 aberrantly regulated AS events in MCF7 breast cancer cells compared to non-tumorigenic epithelial breast MCF10A cells. Though RASL-seq is based on annotated targets without allowing de novo discovery of novel AS events, our results showed that this methodology could provide useful and robust information on splicing errors occurring in breast cancer cells and could be potentially applied to other cancer types.

Notably, GO analysis of differentially spliced genes showed enrichment for biological processes—including cell–cell adhesion, G2/M transition of mitotic cell cycle, regulation of GTPase activity, Wnt signaling pathway, ubiquitin protein ligase binding, protein kinase activity, and regulation of cell growth—directly linked to oncogenesis and cancer progression. For instance, it was previously reported that Wnt signaling is elevated across multiple subtypes of human breast cancer [52] and the Wnt signaling cascade has been involved in the initiation and progression of breast tumors [53].

Interestingly, among validated AS events that are highly dysregulated in cancer cells, we found *ADD3* exon 13, an exon previously identified as being up-regulated in highly metastatic murine breast tumors [54]. Furthermore, we found that genes harboring dysregulated cassette exons have relevant functions in breast cancer and are linked to patient prognosis. Indeed, the identification of protein–protein interaction networks of proteins containing aberrantly regulated AS exons showed the presence of numerous direct interactions with key players of breast tumorigenesis, such as the RHOA GTPase (that interacts with DIAPH1), the AKT activator PIK3CA (that is encoded by the second most frequently mutated gene in breast cancer) [55], or the oncogenic protein kinase Cι (encoded by PRKCI gene).

Importantly, we were able to validate a large fraction of the AS errors occurring in breast cancer cells also in primary breast cancer specimens. In particular, we take advantage of the transcriptomic data of the Cancer Genome Atlas (TCGA), which allow us to investigate splicing profiles in a large number of breast cancer samples (~1000) and non-pathological tissues (~100) as controls, but also by RT-PCR in two normal breast tissues and two breast cancer specimens.

Finally, in our functional analysis of aberrantly expressed AS exons in breast cancer cells and tissues (*PACSIN2* exon 8, *DIAPH1* exon 2, *MARK3* exon 16, *ADD3* exon 13, and *MAP3K7* exon 12), we found that dysregulated cassette exons encode for IDRs. Notably, alternatively spliced exons frequently encode for disordered segments, thus affecting a number of protein functions or properties [43,56]. For instance, IDRs often contain binding sites for other factors, nucleic acids, and small molecules, thus directly affecting the molecular interaction networks of these proteins [43]. Biochemically, IDRs are depleted of bulky hydrophobic residues and enriched of charged and polar amino acids that do not mediate cooperative folding [57]. Sites of post-translational modifications are frequently found in IDRs, thus potentially altering downstream signaling through recruitment of different effectors [43]. Additionally, the flexibility of IDRs could allow conformational heterogeneity between structured domains thus affecting effector signaling.

Notably, we also found that the presence/absence of IDR-encoding AS exons in the *DIAPH1*, *MARK3*, *ADD3*, and *MAP3K7* mRNAs was correlated with gemcitabine sensitivity in a panel of breast cancer cell lines, further supporting the notion that aberrant AS regulation plays a pivotal role in cancer cells drug resistance and/or susceptibility [58].

Even if we cannot assess the molecular mechanisms through which aberrantly regulated AS cassette exons contribute to breast cancer formation and progression, our data further support the relevance of IDR-encoding AS exons in breast cancer cells.

## 5. Conclusions

Collectively, our results expand our knowledge of AS defects occurring in breast cancer cells. In addition to providing a better comprehension of malignant transformation, our analysis identified AS errors that could represent new tools for the diagnosis and classification of cancers or could be used as targets for innovative therapeutical interventions.

## Data Availability

Links to publicly archived datasets analyzed in this study have been provided in the “Material and Methods” section.

## Supplementary Materials

The following are available online at https://www.mdpi.com/article/10.3390/cancers13123071/s1, Figure S1. Validation of AS deregulated cassette exons in MCF10A, ER-positive breast cancer cell lines (MCF7 and T47D), and triple-negative breast cancer cells (MDAMB231 and BT549). Uncropped figures are shown in Supplementary Figure S9; Figure S2. Analysis of protein–protein interaction networks of factors with alternatively spliced cassette exons up-regulated in our analysis. Nodes represent genes of the network. Segments indicate interactions (pink = experimental/biochemical data; aqua green = association in curated database; light violet = protein homology); Figure S3. Analysis of protein–protein interaction networks of factors with alternatively spliced cassette exons down-regulated in our analysis. Nodes represent genes of the network. Segments indicate interactions (pink = experimental/biochemical data; aqua green = association in curated database; light vio-let = protein homology); Figure S4. Validation of TCGA SpliceSeq profiles in human breast cancer samples vs. non-pathological tissues. Quantification of cassette exon inclusion is indicated below each gel. Uncropped figures are shown in Supplementary Figure S10; Figure S5. Correlation of IDR-encoding AS events with chemotherapeutic sensitivity in breast cancer cells. Correlation between indicated alternatively spliced transcripts (% of mRNAs) and sensitivity to paclitaxel (blue), docetaxel (magenta), 5-fluorouracil (green), and tamoxifen (orange) in 41 breast cancer cell lines (AUC, area under the dose–response curve). Pearson coefficient (r) and linear regression are also shown. * *p* < 0.05; Figure S6. Correlation of the PACSIN2 exon 8 encoding an IDR with chemotherapeutic sensitivity in breast cancer cells. Correlation between the alternatively spliced transcripts (% of mRNAs) of PACSIN2 including exon 8 and sensitivity to paclitaxel (blue), docetaxel (magenta), 5-fluorouracil (green), tamoxifen (orange), and gemcitabine (red) sensitivity in 41 breast cancer cell lines (AUC, area under the dose–response curve). Pearson coefficient (r) and linear regression are also shown; Figure S7. Uncropped figures of Figure S3; Figure S8. Uncropped figures of Figure S4; Figure S9. Uncropped figures of Figure S1; Figure S10. Uncropped figures of Figure S4; Table S1. Primers used for RT-PCR and RT-qPCR; Table S2. List of up-, down-, and non-differentially regulated AS events in human breast cancer cells; Table S3. List of gene distributions of regulated AS in human breast cancer cells; Table S4. List of genes in GO analysis; Table S5. Genes with differential AS profile in MCF10A vs. MCF7 cells; Table S6. Protein domains and fraction of disordered residues in selected AS exons identified in our analysis; Table S7. Drug cancer cell line sensitivity (AUC values) to paclitaxel, docetaxel, 5-fluorouracil, tamoxifen, and gemcitabine data. % of transcripts including *ADD3* exon 13, *MAP3K7* exon 12, *DIAPH* exon 2, *PACSIN2* exon 8, and *MARK3* exon 16 for each breast cancer cell line are also indicated.
