# Supplementary material for: Global Alternative Splicing Defects in Human Breast Cancer Cells
Source: Cancers (Basel). 2021 Jun 20;13(12):3071. doi: 10.3390/cancers13123071 (PMC8235023; doi:10.3390/cancers13123071)
Supplement: Supplementary file 1 [file cancers-13-03071-s001.zip › Supplementary figures.pptx]

## Slide 1
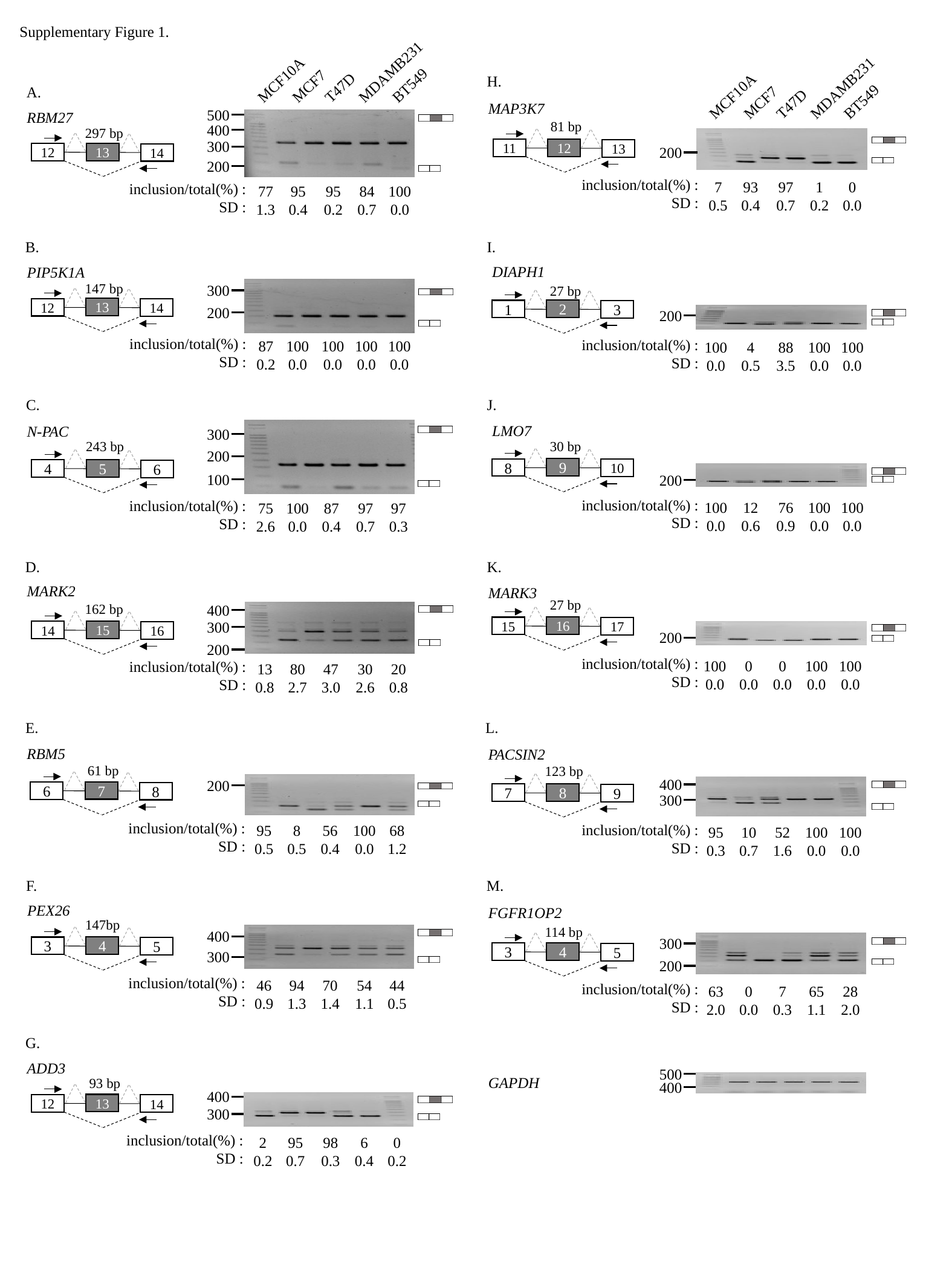

Supplementary Figure 1.
MDAMB231
MCF10A
BT549
MCF7
T47D
A.
500
400
300
200
RBM27
297 bp
13
12
14
inclusion/total(%) :
SD :
77
1.3
95
0.4
95
0.2
84
0.7
100
0.0
H.
MAP3K7
81 bp
12
11
13
200
inclusion/total(%) :
SD :
7
0.5
93
0.4
97
0.7
1
0.2
0
0.0
MDAMB231
MCF10A
BT549
MCF7
T47D
B.
PIP5K1A
147 bp
13
12
14
300
200
inclusion/total(%) :
SD :
87
0.2
100
0.0
100
0.0
100
0.0
100
0.0
I.
 DIAPH1
27 bp
2
1
3
200
inclusion/total(%) :
SD :
100
0.0
4
0.5
88
3.5
100
0.0
100
0.0
C.
N-PAC
243 bp
5
4
6
300
200
100
inclusion/total(%) :
SD :
75
2.6
100
0.0
87
0.4
97
0.7
97
0.3
J.
 LMO7
30 bp
9
8
10
200
inclusion/total(%) :
SD :
100
0.0
12
0.6
76
0.9
100
0.0
100
0.0
D.
MARK2
162 bp
15
14
16
400
300
200
inclusion/total(%) :
SD :
13
0.8
80
2.7
47
3.0
30
2.6
20
0.8
K.
MARK3
27 bp
16
15
17
200
inclusion/total(%) :
SD :
100
0.0
0
0.0
0
0.0
100
0.0
100
0.0
E.
RBM5
61 bp
7
6
8
200
inclusion/total(%) :
SD :
95
0.5
8
0.5
56
0.4
100
0.0
68
1.2
L.
PACSIN2
123 bp
8
7
9
400
300
inclusion/total(%) :
SD :
95
0.3
10
0.7
52
1.6
100
0.0
100
0.0
F.
PEX26
147bp
4
3
5
400
300
inclusion/total(%) :
SD :
46
0.9
94
1.3
70
1.4
54
1.1
44
0.5
M.
FGFR1OP2
114 bp
300
4
3
5
200
inclusion/total(%) :
SD :
63
2.0
0
0.0
7
0.3
65
1.1
28
2.0
G.
ADD3
93 bp
13
12
14
400
300
inclusion/total(%) :
SD :
2
0.2
95
0.7
98
0.3
6
0.4
0
0.2
500
GAPDH
400

## Slide 2
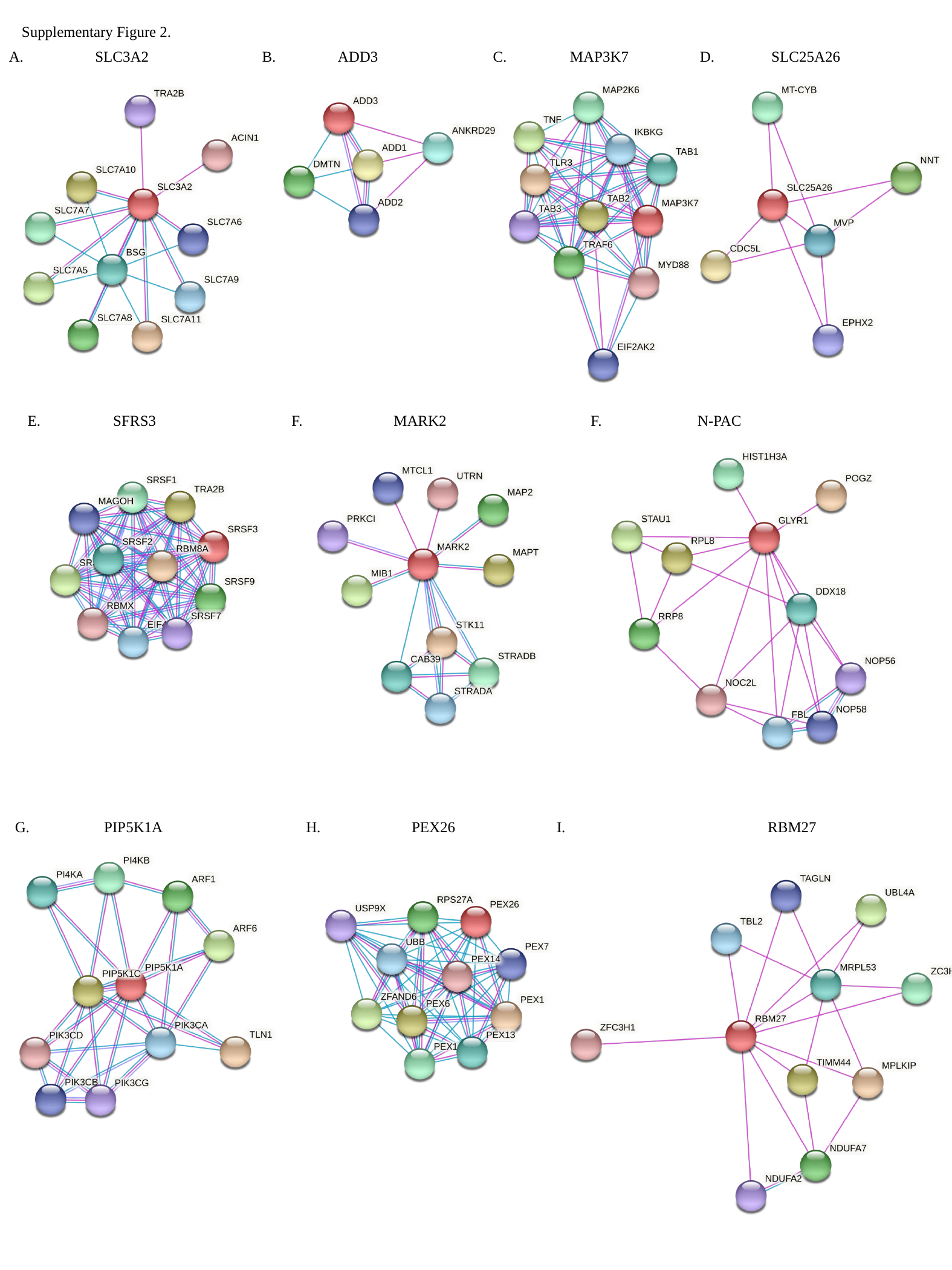

Supplementary Figure 2.
A.
SLC3A2
B.
ADD3
C.
MAP3K7
D.
SLC25A26
E.
SFRS3
F.
MARK2
F.
N-PAC
G.
PIP5K1A
H.
PEX26
I.
RBM27

## Slide 3
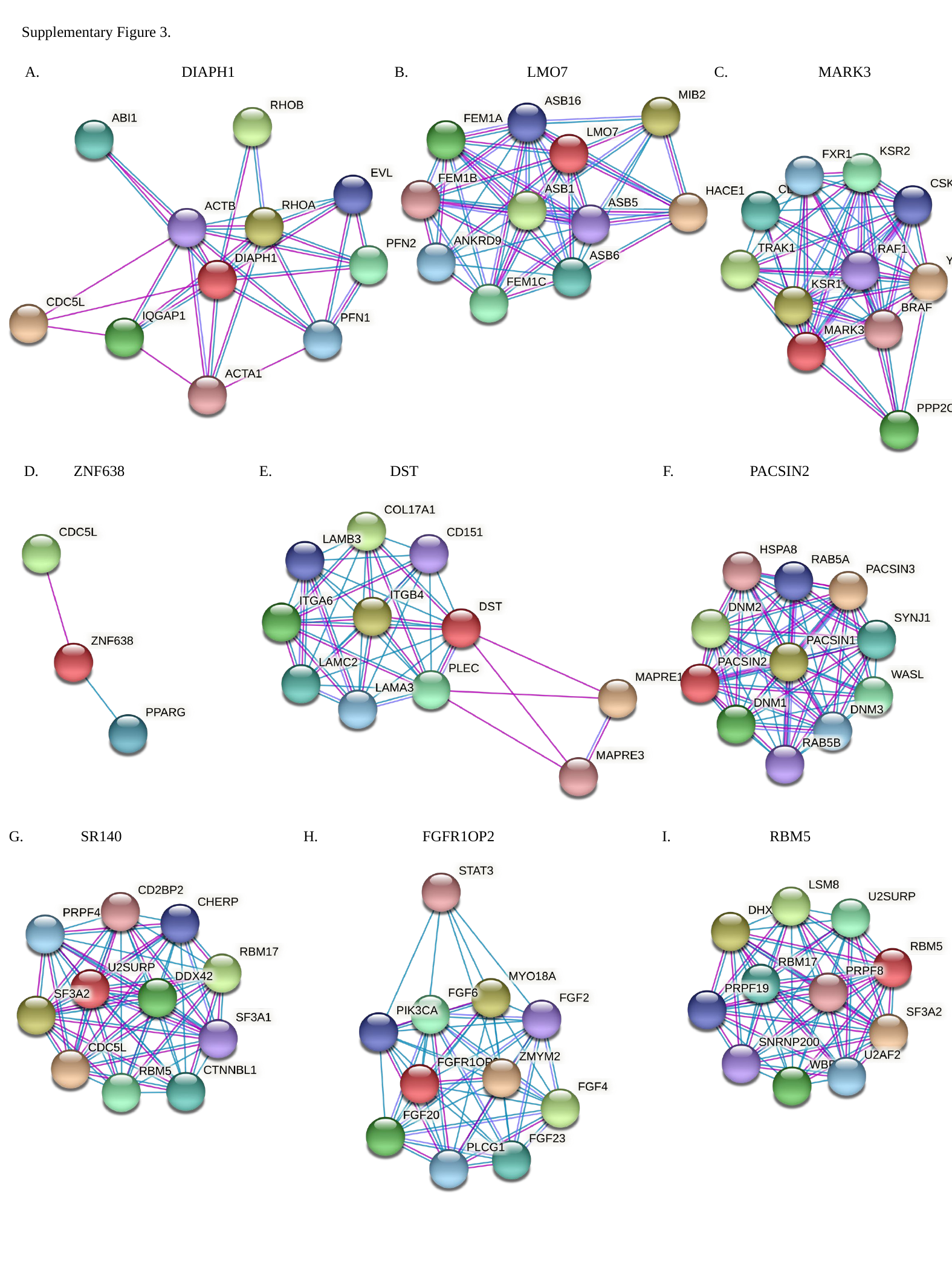

Supplementary Figure 3.
A.
 DIAPH1
B.
 LMO7
C.
MARK3
D.
ZNF638
E.
DST
F.
PACSIN2
G.
SR140
H.
FGFR1OP2
I.
RBM5

## Slide 4
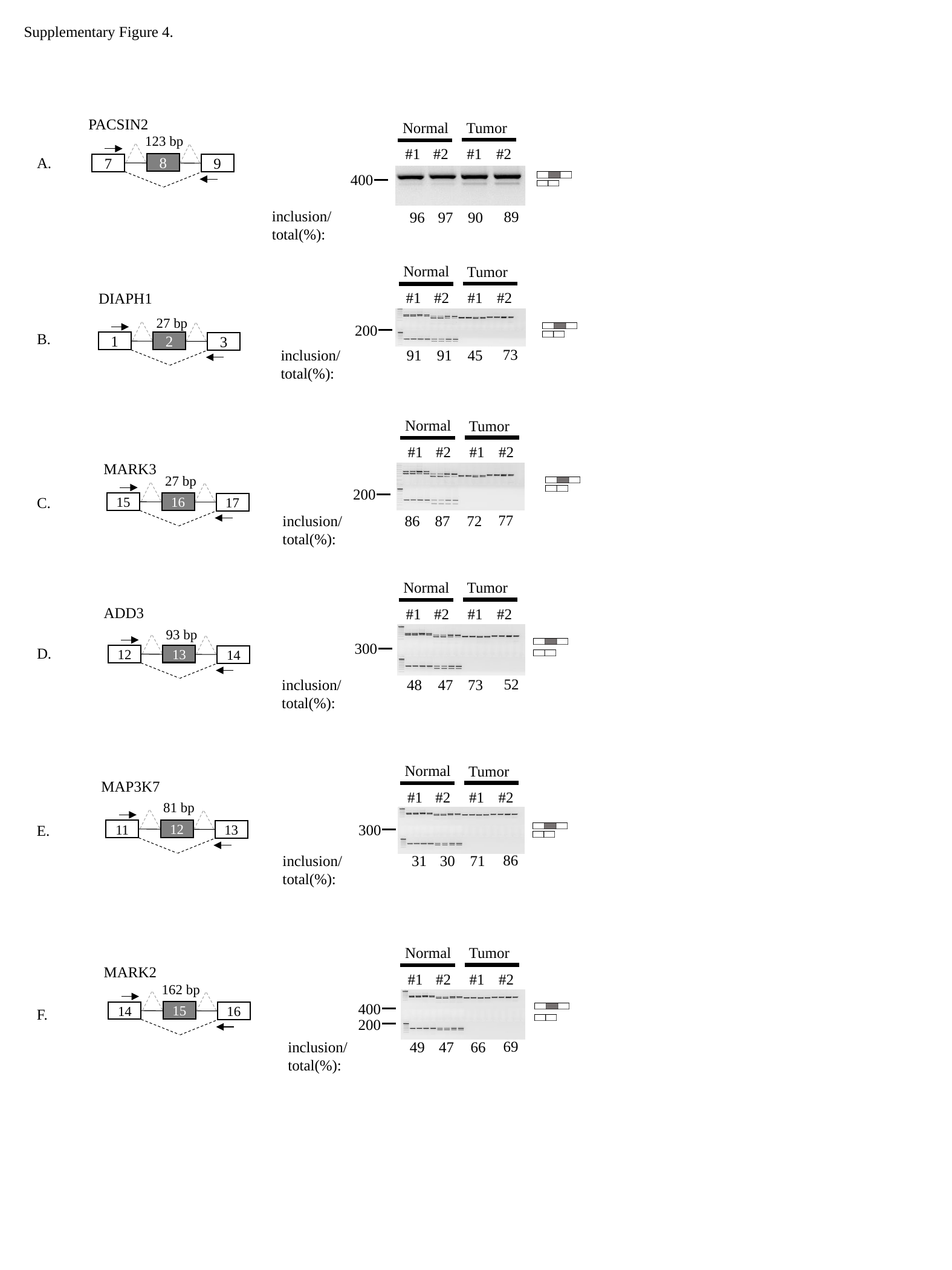

Supplementary Figure 4.
PACSIN2
Normal
Tumor
#1
#2
#1
#2
123 bp
8
7
9
A.
400
inclusion/total(%):
89
96
97
90
Normal
Tumor
#1
#2
#1
#2
 DIAPH1
27 bp
200
2
1
3
B.
73
91
91
45
inclusion/total(%):
Normal
Tumor
#1
#2
#1
#2
MARK3
27 bp
200
16
15
17
C.
77
86
87
72
inclusion/total(%):
Normal
Tumor
#1
#2
#1
#2
ADD3
93 bp
300
13
12
14
D.
52
48
47
73
inclusion/total(%):
Normal
Tumor
#1
#2
#1
#2
MAP3K7
81 bp
12
11
13
300
E.
86
31
30
71
inclusion/total(%):
Normal
Tumor
#1
#2
#1
#2
MARK2
162 bp
15
14
16
400
F.
200
69
49
47
66
inclusion/total(%):

## Slide 5
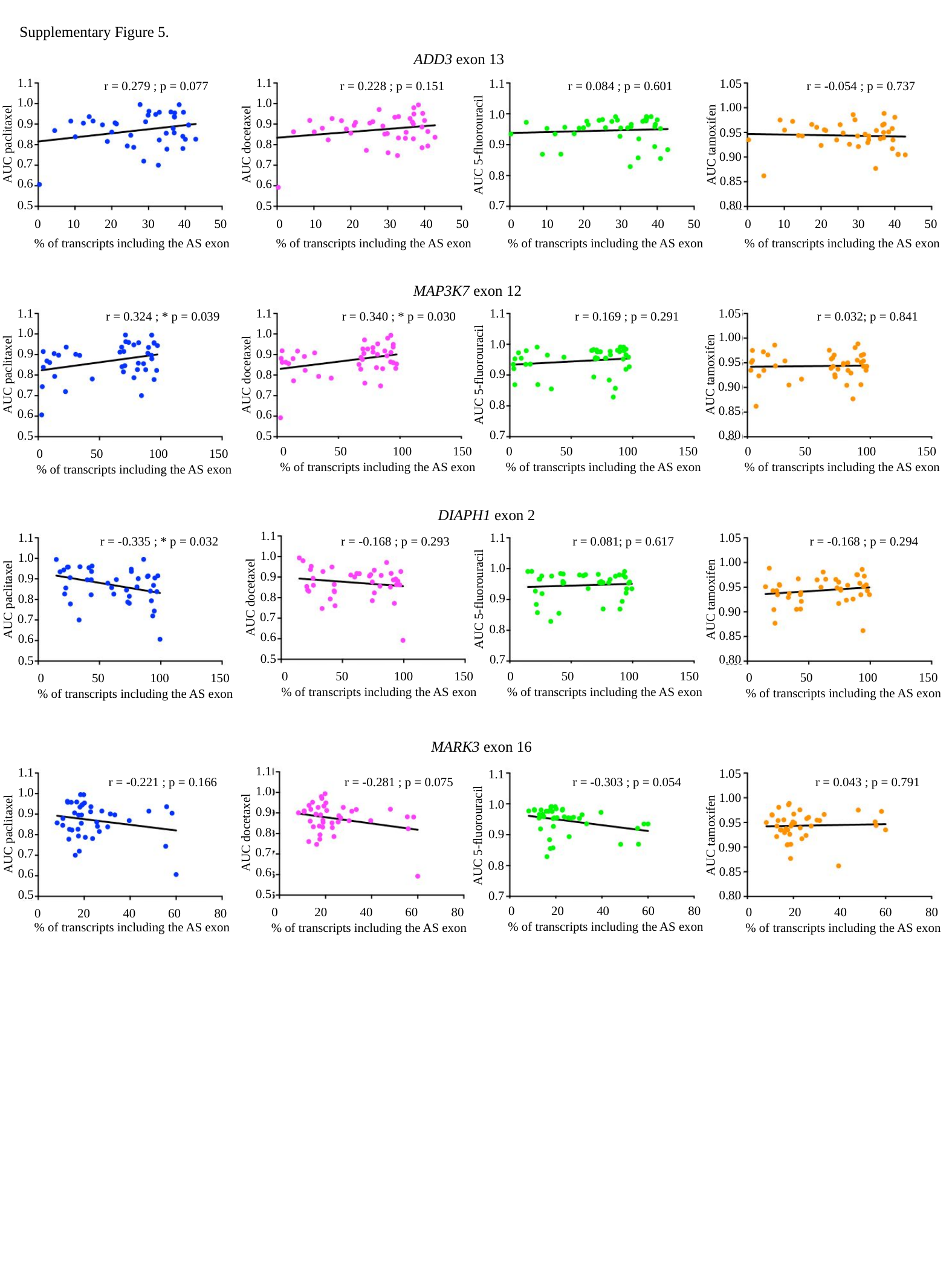

Supplementary Figure 5.
ADD3 exon 13
1.1
1.1
1.05
1.1
r = 0.279 ; p = 0.077
r = 0.228 ; p = 0.151
r = 0.084 ; p = 0.601
r = -0.054 ; p = 0.737
1.0
1.0
1.00
1.0
0.9
0.9
0.95
0.8
0.9
0.8
AUC paclitaxel
AUC docetaxel
AUC tamoxifen
AUC 5-fluorouracil
0.90
0.7
0.7
0.8
0.85
0.6
0.6
0.80
0.5
0.7
0.5
0
10
20
30
40
50
0
10
20
30
40
50
0
10
20
30
40
50
0
10
20
30
40
50
% of transcripts including the AS exon
% of transcripts including the AS exon
% of transcripts including the AS exon
% of transcripts including the AS exon
MAP3K7 exon 12
1.1
1.05
1.1
1.1
r = 0.324 ; * p = 0.039
r = 0.340 ; * p = 0.030
r = 0.169 ; p = 0.291
r = 0.032; p = 0.841
1.0
1.0
1.00
1.0
0.9
0.9
0.95
0.9
0.8
0.8
AUC paclitaxel
AUC tamoxifen
AUC docetaxel
AUC 5-fluorouracil
0.90
0.7
0.7
0.8
0.85
0.6
0.6
0.80
0.7
0.5
0.5
0
50
100
150
0
50
100
150
0
50
100
150
0
50
100
150
% of transcripts including the AS exon
% of transcripts including the AS exon
% of transcripts including the AS exon
% of transcripts including the AS exon
DIAPH1 exon 2
1.1
1.1
1.1
1.05
r = -0.335 ; * p = 0.032
r = -0.168 ; p = 0.293
r = 0.081; p = 0.617
r = -0.168 ; p = 0.294
1.0
1.0
1.00
1.0
0.9
0.9
0.95
0.8
0.9
AUC docetaxel
0.8
AUC 5-fluorouracil
AUC paclitaxel
AUC tamoxifen
0.90
0.7
0.7
0.8
0.85
0.6
0.6
0.5
0.7
0.80
0.5
0
50
100
150
0
50
100
150
0
50
100
150
0
50
100
150
% of transcripts including the AS exon
% of transcripts including the AS exon
% of transcripts including the AS exon
% of transcripts including the AS exon
MARK3 exon 16
1.1
1.1
1.05
1.1
r = -0.221 ; p = 0.166
r = -0.281 ; p = 0.075
r = -0.303 ; p = 0.054
r = 0.043 ; p = 0.791
1.0
1.0
1.00
1.0
0.9
0.9
0.95
0.8
AUC docetaxel
0.8
0.9
AUC paclitaxel
AUC tamoxifen
AUC 5-fluorouracil
0.90
0.7
0.7
0.8
0.85
0.6
0.6
0.5
0.5
0.80
0.7
0
20
40
60
80
0
20
40
60
80
0
20
40
60
80
0
20
40
60
80
% of transcripts including the AS exon
% of transcripts including the AS exon
% of transcripts including the AS exon
% of transcripts including the AS exon

## Slide 6
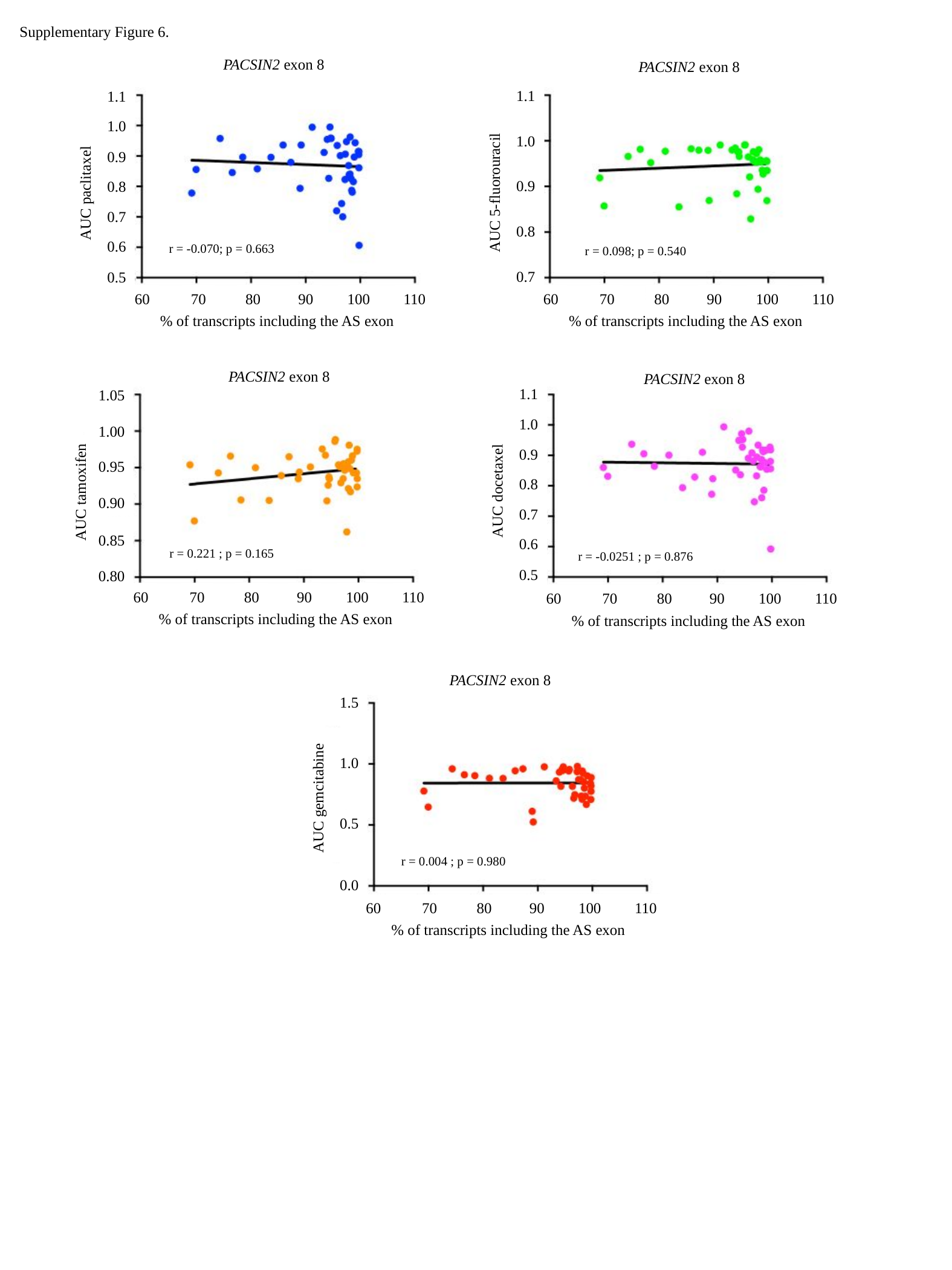

Supplementary Figure 6.
PACSIN2 exon 8
PACSIN2 exon 8
1.1
1.1
1.0
1.0
0.9
0.9
0.8
AUC 5-fluorouracil
AUC paclitaxel
0.7
0.8
0.6
r = -0.070; p = 0.663
r = 0.098; p = 0.540
0.7
0.5
100
110
100
110
60
70
80
90
60
70
80
90
% of transcripts including the AS exon
% of transcripts including the AS exon
PACSIN2 exon 8
PACSIN2 exon 8
1.1
1.05
1.0
1.00
0.9
0.95
0.8
AUC docetaxel
AUC tamoxifen
0.90
0.7
0.85
0.6
r = 0.221 ; p = 0.165
r = -0.0251 ; p = 0.876
0.5
0.80
100
110
60
70
80
90
100
110
60
70
80
90
% of transcripts including the AS exon
% of transcripts including the AS exon
PACSIN2 exon 8
1.5
1.0
AUC gemcitabine
0.5
r = 0.004 ; p = 0.980
0.0
100
110
60
70
80
90
% of transcripts including the AS exon

## Slide 7
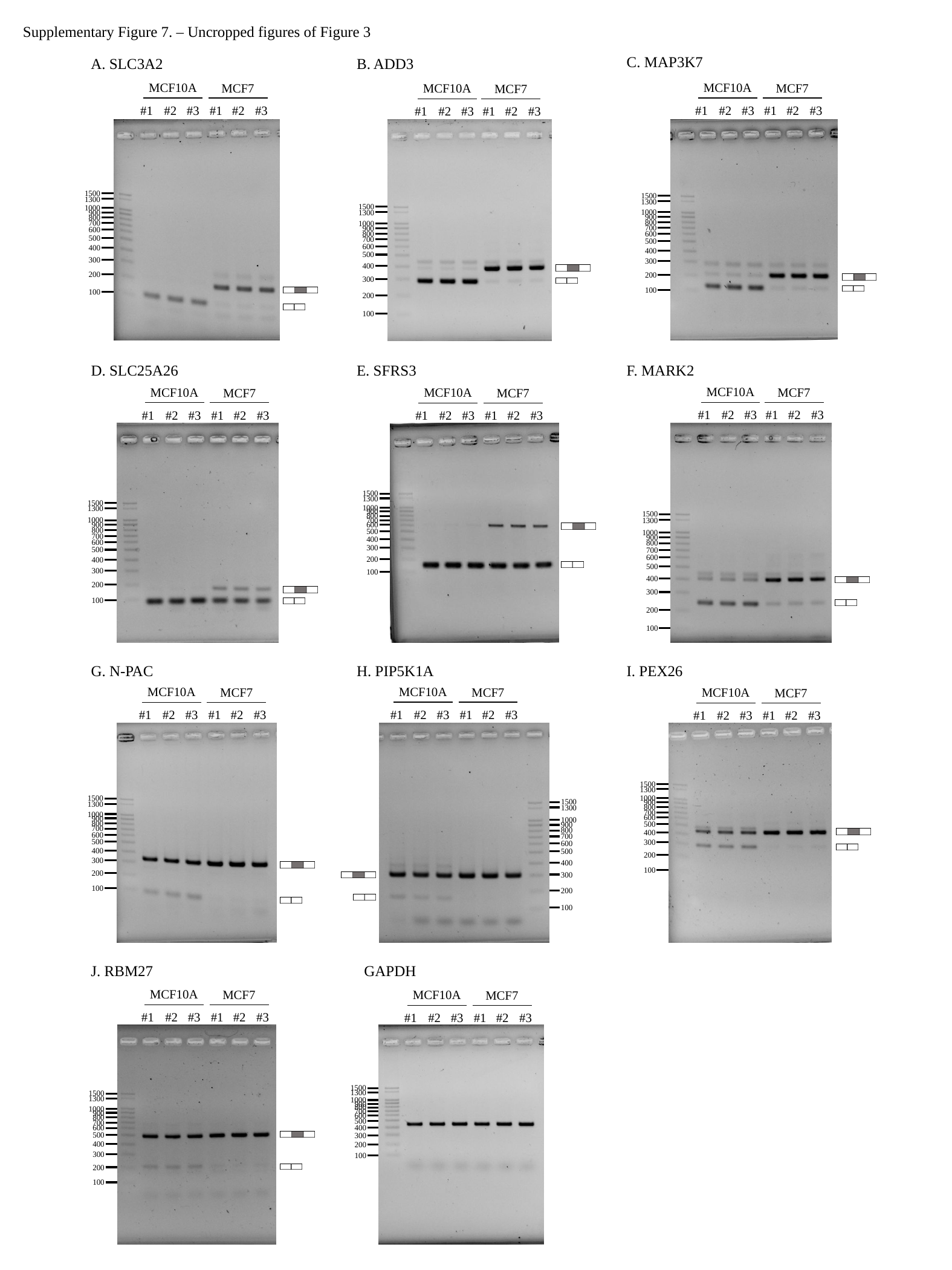

Supplementary Figure 7. – Uncropped figures of Figure 3
C. MAP3K7
A. SLC3A2
B. ADD3
MCF10A
MCF10A
MCF7
MCF7
MCF10A
MCF7
#1
#2
#3
#1
#2
#3
#1
#2
#3
#1
#2
#3
#1
#2
#3
#1
#2
#3
1500
1500
1300
1300
1500
1000
1000
1300
900
900
800
800
700
1000
700
900
600
800
600
500
700
500
600
400
400
500
300
300
400
200
200
300
100
100
200
100
D. SLC25A26
E. SFRS3
F. MARK2
MCF10A
MCF10A
MCF7
MCF10A
MCF7
MCF7
#1
#2
#3
#1
#2
#3
#1
#2
#3
#1
#2
#3
#1
#2
#3
#1
#2
#3
1500
1300
1500
1000
1300
900
1500
800
1000
1300
700
900
600
800
500
1000
700
900
400
600
800
300
500
700
600
200
400
500
300
100
400
200
300
100
200
100
G. N-PAC
H. PIP5K1A
I. PEX26
MCF10A
MCF10A
MCF7
MCF10A
MCF7
MCF7
#1
#2
#3
#1
#2
#3
#1
#2
#3
#1
#2
#3
#1
#2
#3
#1
#2
#3
1500
1300
1000
1500
1500
900
1300
800
1300
700
1000
600
900
1000
800
500
900
700
800
400
600
700
500
300
600
400
500
200
300
400
100
200
300
100
200
100
J. RBM27
GAPDH
MCF10A
MCF10A
MCF7
MCF7
#1
#2
#3
#1
#2
#3
#1
#2
#3
#1
#2
#3
1500
1300
1500
1300
1000
900
800
1000
700
900
600
800
500
700
400
600
500
300
400
200
300
100
200
100

## Slide 8
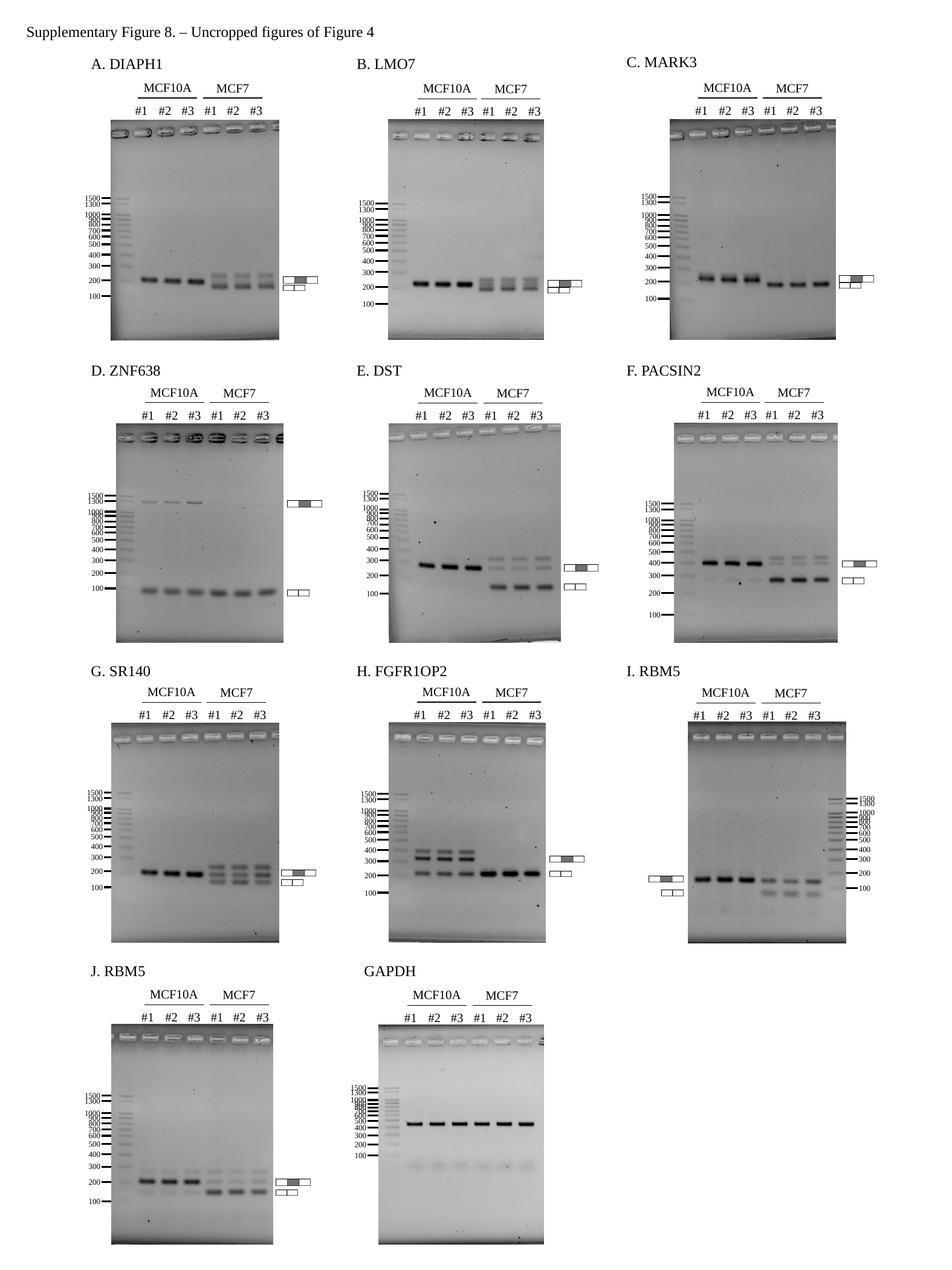

Supplementary Figure 8. – Uncropped figures of Figure 4
C. MARK3
A. DIAPH1
B. LMO7
MCF10A
MCF10A
MCF7
MCF7
MCF10A
MCF7
#1
#2
#3
#1
#2
#3
#1
#2
#3
#1
#2
#3
#1
#2
#3
#1
#2
#3
1500
1500
1300
1500
1300
1300
1000
1000
900
900
1000
800
900
800
800
700
700
700
600
600
600
500
500
500
400
400
400
300
300
300
200
200
200
100
100
100
D. ZNF638
E. DST
F. PACSIN2
MCF10A
MCF10A
MCF7
MCF10A
MCF7
MCF7
#1
#2
#3
#1
#2
#3
#1
#2
#3
#1
#2
#3
#1
#2
#3
#1
#2
#3
1500
1500
1300
1300
1500
1000
1300
1000
900
900
800
1000
800
700
900
700
600
800
600
700
500
500
600
400
400
500
300
300
400
200
200
300
100
200
100
100
G. SR140
H. FGFR1OP2
I. RBM5
MCF10A
MCF10A
MCF7
MCF10A
MCF7
MCF7
#1
#2
#3
#1
#2
#3
#1
#2
#3
#1
#2
#3
#1
#2
#3
#1
#2
#3
1500
1500
1300
1500
1300
1300
1000
1000
1000
900
900
900
800
800
800
700
700
700
600
600
600
500
500
500
400
400
400
300
300
300
200
200
200
100
100
100
J. RBM5
GAPDH
MCF10A
MCF10A
MCF7
MCF7
#1
#2
#3
#1
#2
#3
#1
#2
#3
#1
#2
#3
1500
1300
1500
1000
1300
900
800
700
1000
600
900
500
800
400
700
300
600
500
200
400
100
300
200
100

## Slide 9
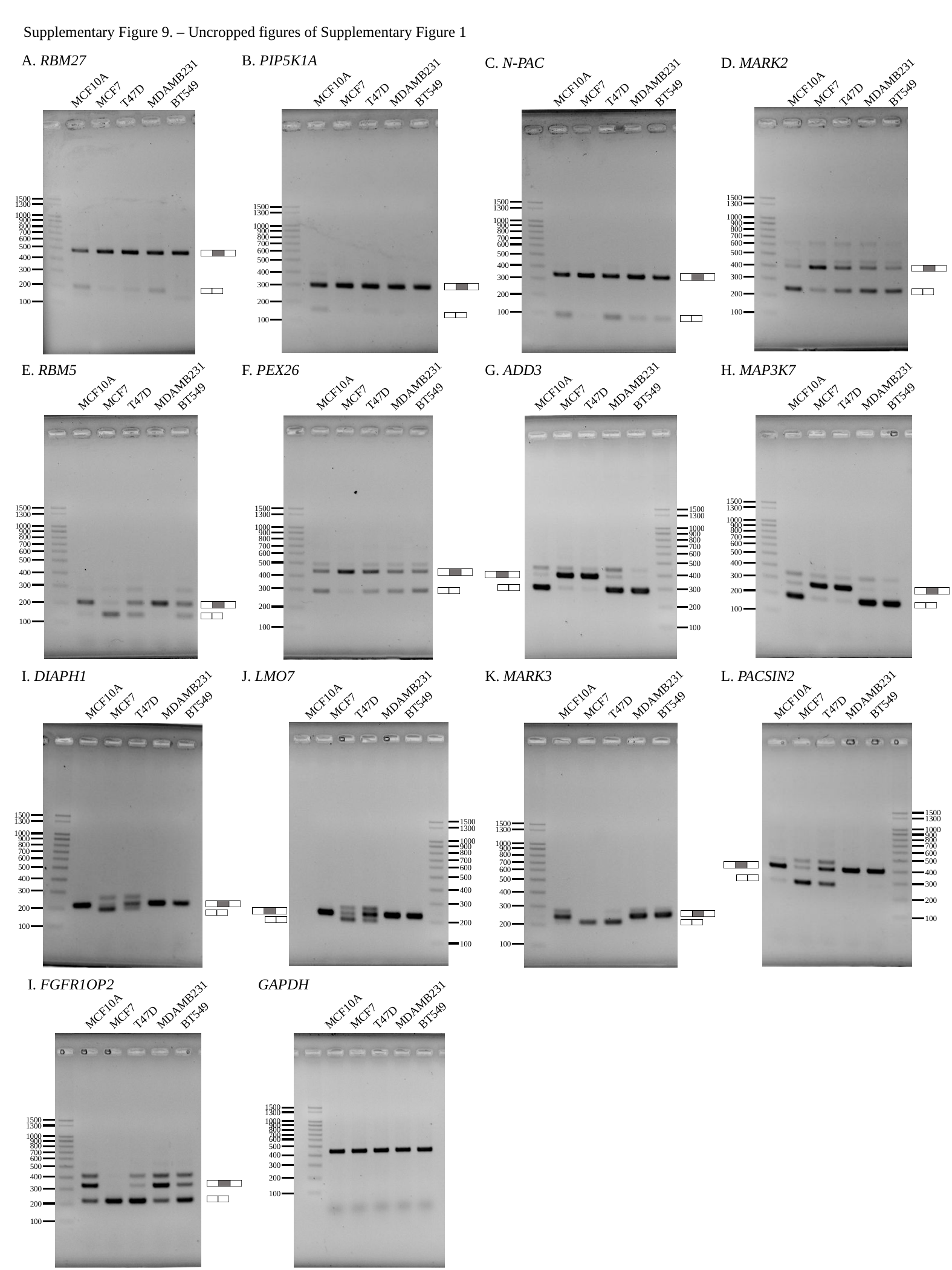

Supplementary Figure 9. – Uncropped figures of Supplementary Figure 1
A. RBM27
B. PIP5K1A
C. N-PAC
D. MARK2
MDAMB231
MDAMB231
MDAMB231
MDAMB231
MCF10A
MCF10A
MCF10A
MCF10A
BT549
BT549
BT549
MCF7
MCF7
MCF7
BT549
T47D
T47D
T47D
MCF7
T47D
1500
1500
1500
1300
1300
1500
1300
1300
1000
1000
900
1000
900
900
1000
800
800
900
800
700
700
800
700
600
600
700
600
500
600
500
500
400
500
400
400
300
400
300
300
200
300
200
200
200
100
100
100
100
E. RBM5
F. PEX26
G. ADD3
H. MAP3K7
MDAMB231
MDAMB231
MDAMB231
MDAMB231
MCF10A
MCF10A
MCF10A
MCF10A
BT549
BT549
BT549
BT549
MCF7
MCF7
MCF7
MCF7
T47D
T47D
T47D
T47D
1500
1300
1500
1500
1500
1300
1300
1300
1000
900
1000
1000
1000
800
900
900
900
700
800
800
800
600
700
700
700
600
500
600
600
500
500
400
500
400
400
400
300
300
300
300
200
200
200
200
100
100
100
100
I. DIAPH1
J. LMO7
K. MARK3
L. PACSIN2
MDAMB231
MDAMB231
MDAMB231
MDAMB231
MCF10A
MCF10A
MCF10A
MCF10A
BT549
BT549
BT549
BT549
MCF7
MCF7
MCF7
MCF7
T47D
T47D
T47D
T47D
1500
1500
1300
1300
1500
1500
1300
1000
1300
1000
900
900
800
1000
1000
800
700
900
900
700
800
600
800
600
700
500
700
500
600
600
400
500
400
500
300
400
300
400
200
300
300
200
100
200
200
100
100
100
I. FGFR1OP2
GAPDH
MDAMB231
MDAMB231
MCF10A
MCF10A
BT549
BT549
MCF7
MCF7
T47D
T47D
1500
1300
1500
1000
900
1300
800
700
1000
600
900
800
500
700
400
600
300
500
400
200
300
100
200
100

## Slide 10
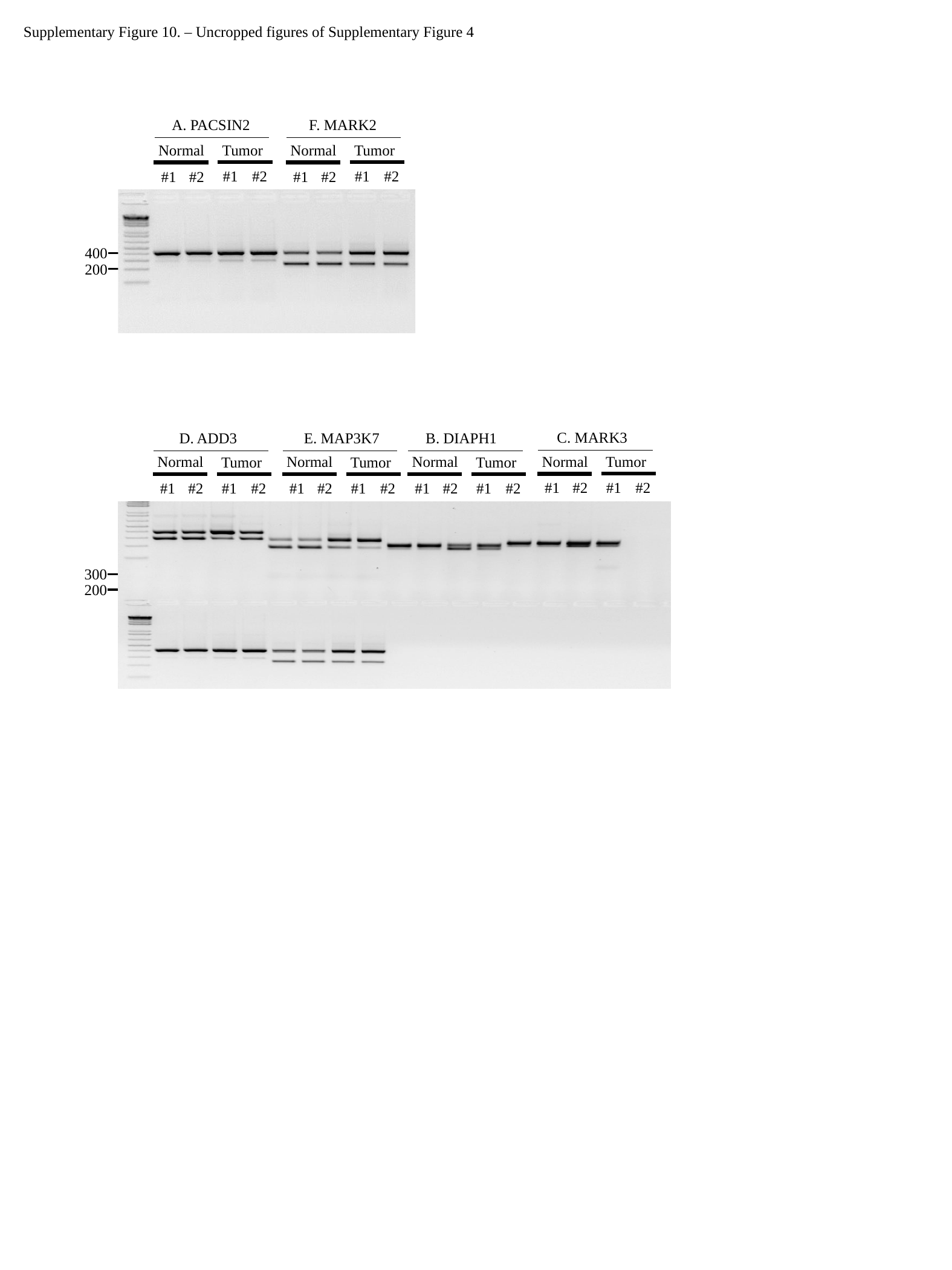

Supplementary Figure 10. – Uncropped figures of Supplementary Figure 4
A. PACSIN2
F. MARK2
Normal
Tumor
#1
#2
#1
#2
Normal
Tumor
#1
#2
#1
#2
400
200
C. MARK3
D. ADD3
E. MAP3K7
B. DIAPH1
Normal
Tumor
#1
#2
#1
#2
Normal
Tumor
#1
#2
#1
#2
Normal
Tumor
#1
#2
#1
#2
Normal
Tumor
#1
#2
#1
#2
300
200
